# Design and development of exome capture sequencing for the domestic pig (*Sus scrofa*)

**DOI:** 10.1186/1471-2164-15-550

**Published:** 2014-07-03

**Authors:** Christelle Robert, Pablo Fuentes-Utrilla, Karen Troup, Julia Loecherbach, Frances Turner, Richard Talbot, Alan L Archibald, Alan Mileham, Nader Deeb, David A Hume, Mick Watson

**Affiliations:** The Roslin Institute and Royal (Dick) School of Veterinary Studies, University of Edinburgh, Easter Bush, Edinburgh, EH25 9RG UK; Edinburgh Genomics, University of Edinburgh, Easter Bush, Edinburgh, EH25 9RG UK; Genus plc, 1525 River Road, DeForest, WI 53532 USA; Genus plc, 100 Bluegrass Commons Blvd. Suite 2200, Hendersonville, TN 37075 USA

## Abstract

**Background:**

The domestic pig (Sus scrofa) is both an important livestock species and a model for biomedical research. Exome sequencing has accelerated identification of protein-coding variants underlying phenotypic traits in human and mouse. We aimed to develop and validate a similar resource for the pig.

**Results:**

We developed probe sets to capture pig exonic sequences based upon the current Ensembl pig gene annotation supplemented with mapped expressed sequence tags (ESTs) and demonstrated proof-of-principle capture and sequencing of the pig exome in 96 pigs, encompassing 24 capture experiments. For most of the samples at least 10x sequence coverage was achieved for more than 90% of the target bases. Bioinformatic analysis of the data revealed over 236,000 high confidence predicted SNPs and over 28,000 predicted indels.

**Conclusions:**

We have achieved coverage statistics similar to those seen with commercially available human and mouse exome kits. Exome capture in pigs provides a tool to identify coding region variation associated with production traits, including loss of function mutations which may explain embryonic and neonatal losses, and to improve genomic assemblies in the vicinity of protein coding genes in the pig.

**Electronic supplementary material:**

The online version of this article (doi:10.1186/1471-2164-15-550) contains supplementary material, which is available to authorized users.

## Background

Reductions in the cost of DNA sequencing [1], have brought the large scale sequencing of individual human genomes within reach financially and there are claims that the $1000 human genome is now achievable. Low cost sequencing has facilitated the sequencing of multiple human genomes and analysis of these genomes has revealed that most individuals harbour hundreds of deleterious mutations [2]. In humans, approximately 85% of known disease-causing mutations can be found within the coding region or splice sites of protein-coding genes [3]. Whilst this number may be biased by studies focused only on protein-coding genes, exome sequencing has nevertheless become a standard tool in the search for the cause of monogenic disorders in humans [3–7]. Exome capture was initially carried out using microarrays [8, 9] but current methods, such as Agilent’s SureSelect and Nimblegen’s SeqCap EZ systems rely on liquid capture [10, 11].

Global pig meat production has risen by 60% in the last decade and now accounts for approximately one third of all meat produced worldwide. One of the key aims of farm animal genetics and genomics research is to discover the genes underlying important traits such as disease resistance/susceptibility, feed efficiency, product quality and reproductive performance. Whereas many quantitative traits are linked to variation in gene expression, the initial sequencing of the pig genome revealed that individual pigs also harbour significant numbers of potentially deleterious protein-coding mutations [12].

As has been noted in the recent identification of the lethal recessive gene in Holstein cattle that causes brachyspina [13], and a mutation in the CWC15 gene in Jersey cattle associated with reduced fertility [14], such mutations can be present at high frequency in livestock populations and lead to significant fetal loss. At least some of the large numbers of pre-natal and pre-weaning embryo losses in pigs (up to 40% [15]) are also likely to be due to homozygous lethal mutations.

As well as being economically important in its own right, the pig is an important model for human disease [16, 17]. Pigs resemble humans in terms of development and physiology, and pig and human genomes are much more closely related than the human and mouse [12, 18]. Pigs are also more closely related to humans in terms of their innate immune-responses [19]. The combination of the rapid identification of natural null mutations by exome sequence analyses along with the option to establish brother-sister mating in a multiparous animal will facilitate the development of models based on homozygous natural null mutations. Such models will be valuable for basic and strategic biological and biomedical research.

Here we describe the design and development of an exome capture for the domestic pig Sus scrofa, based on the Ensembl [20] annotation of assembly version 10.2 of the genome [12]. We have augmented the design for the capture probe set with additional known expressed sequences from UniGene [21]. We provide comprehensive statistics from a pilot study in which the exomes of 96 pigs were sequenced using the Roche Nimblegen SeqCAP EZ system and the Illumina HiSeq 2500 platform. Our bioinformatic analyses, using variant discovery pipelines based on GATK [22], revealed several hundred thousand putative high quality variants within our capture region, demonstrating the power of exome sequencing. The results indicate that each individual pig harbours several thousand non-synonymous amino acid substitutions.

## Results

### Definition of the capture region

**Figure 1:**
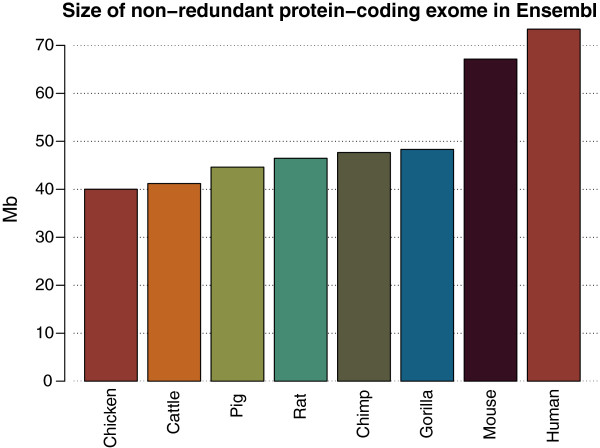
**Comparison of exome regions.** The size of the core Ensembl protein-coding exome in a range of species, including human, important agricultural species (chicken, cattle, pig); important rodent model species (rat, mouse); and important primate (gorilla, chimp) species.

The exons of protein coding genes from release 71 of the Ensembl genebuild for Sus scrofa sum to a total length of 44.6 Mb, compared to 73.4 Mb for human and 67.1 Mb for mouse (Figure 1). These figures for the human and murine genomes exclude alternative haplotypes. Assuming that pig does not have a smaller exome than mouse or human, there is probably 22.5 to 28.8 Mb missing from the current pig genome assembly and Ensembl annotation of the current pig genome. By mapping publicly available expressed sequence tags (ESTs) to the reference genome, we were able to identify expressed regions that were not covered by the Ensembl gene set. The “ensembl + ests” capture region covers 58.1 Mb, suggesting there is still between 9 and 15.3 Mb missing once additional EST alignments have been taken into account.

**Figure 2:**
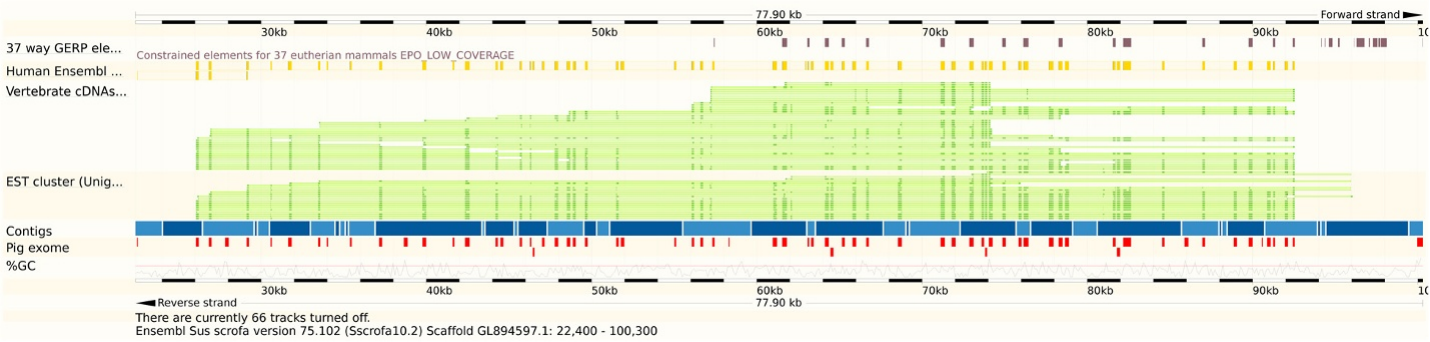
**Additional gene: putative SMG1 phosphatidylinositol 3-kinase.** Genomic region displayed in Ensembl with good alignment to a UniGene mRNA sequence representing an 11 kb putative SMG1 phosphatidylinositol 3-kinase-related sequence which is homologous to the human SMG1 gene. Alignments to several cDNA and EST sequences are annotated in Ensembl in this region (22,400-100,300 bp) on unplaced scaffold GL894597.1 but no Ensembl gene model has been built or core gene defined. The red regions show the pig exome capture regions described in this paper, derived from alignments of UniGene sequences against the genome (see “Methods”). NCBI annotation of the pig genome includes a gene model for SMG1 at this location.

**Figure 3:**
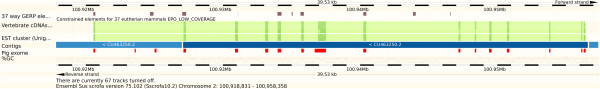
**Additional gene: putative GPCR98.** Genomic region displayed in Ensembl with good alignment to a UniGene mRNA sequence representing an 8.7 kb putative GPCR98 mRNA. Alignments to cDNA and EST sequences are annotated in Ensembl in this region (100,918,831-100,958,358 bp) on chromosome 2 but no Ensembl gene model has been built or core gene defined. The red regions show the pig exome capture regions described in this paper, derived from alignments of UniGene sequences against the genome (see “Methods”).

Based upon mapping of the ESTs and cDNAs from UniGene to the current pig reference genome in Ensembl, we were able to identify two classes of gene: (i) those genes present in the pig assembly, but either wholly or partially absent in the Ensembl gene build; and (ii) those genes completely absent from the assembly.Figures 2 and 3 show examples where very good alignments of ESTs to the genome are achievable but have not been included in the core Ensembl gene set. The region in Figure 2 shows an alignment against the latest pig reference of an 11 kb Sus scrofa mRNA from UniGene, a putative SMG1 phosphatidylinositol 3-kinase-related kinase homologous to the human SMG1 gene. Alignments to several cDNA and EST sequences are annotated in Ensembl in this region (22,400-100,300 bp) on unplaced scaffold GL894597.1 but no Ensembl gene model has been built or core gene defined. A gene model for the ARL6IP1 gene is annotated on this scaffold in the adjacent 10 kb interval (GL894597.1:100000–111000). The human homologues of SMG1 and ARL6IP1 are co-located in a 140 kb interval on human chromosome 16. NCBI annotation of the pig genome includes a gene model for SMG1 at this location. Similarly, Figure 3 shows a region of pig chromosome 2 (2:100,918,831-100,958,358 bp) matching an 8.7 kb mRNA from UniGene, which represents a putative G protein-coupled receptor 98 (GPCR 98) mRNA. NCBI annotation of the pig genome includes a gene model for LOC100519889 G-protein coupled receptor 98-like at this location. In humans the GPR98 gene with >90 exons and 11 annotated transcripts spans 635 kb on human chromosome 5 and is flanked by the LYSMD3 and ARRDC3 genes. In the homologous interval between the LYSDM3 and ARRDC3 loci on pig chromosome 2 a small GPR98 gene model with 3 exons spanning ~13 kb is annotated in Ensembl. Hence the Ensembl pig GPR98 gene model probably represents only a fraction of the pig GPR98 exons and gene.

**Figure 4:**
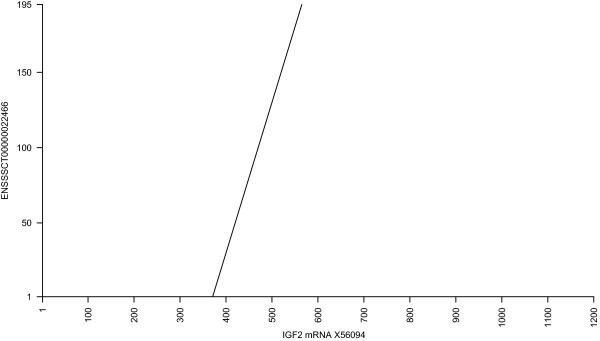
**Dot plot comparison of Ensembl and EMBL/GenBank IGF2 mRNA sequences.** A dot plot comparison of Ensembl transcript ENSSSCT00000022466 and sequence accession X56094, both of which represent IGF2. Clearly, ENSSSCT00000022466 represents only a fragment of the full length mRNA.

Anther gene only partially represented in the Ensembl gene build is IGF2 (insulin-like growth factor 2), which plays a key role in mammalian growth and development [23]. Although the pig IGF2 gene has been characterised in detail including the identification of a point mutation with significant effects on muscling [24], there is only a fragment of the gene in the published pig genome sequence (Sscrofa10.2; [12]). In Ensembl version 71, this gene is represented by transcript ENSSSCT00000022466, consisting of 195 bp. The corresponding genomic region is represented by multiple small contigs separated by gaps. In contrast, sequence accession X56094 is a 1225 bp mRNA sequence representing the Sus scrofa IGF2. Figure 4 shows a dot-plot comparing these two sequences, and demonstrates that ENSSSCT00000022466 represents only a fragment of the full-length IGF2 mRNA.

In total, we aligned 50,106 publicly available mRNA and EST sequences from Sus scrofa Unigene to the latest pig genome build, and added 39,635 additional high scoring alignments to the proposed capture region before merging and filtering the dataset (see “Methods”). A further 95 full length mRNA sequences were not detectable at all in the assembly, and these were added to the target capture as FASTA sequences. These are listed in Additional file 1: Table S1.

### Probe design and size of the final target region

Target genomic regions were provided in BED format to Roche Nimblegen for probe design. Any regions less than 100 bp in size were padded equally at both ends to become 100 bp in length. Any genomic regions found to overlap were subsequently merged to form one continuous region. Probes were designed to the resulting capture regions with an additional 100 bp offset. Only those probes that had high-scoring matches to less than 6 genomic regions were used in the capture (see Methods). The probe design resulted in a 60.6 Mb target, with probes covering 98.4% of the bases in those regions. The design is available through Roche Nimblegen, design name 130104_Sscr_10_2_MW_EZ_HX1.

### Sequence and coverage statistics

We captured and sequenced exomes from a total of 96 pigs, in 16 pools of 6 individual pig DNA samples from unrelated commercial pigs, using the Illumina HiSeq 2500 platform. The animals whose exomes were sequenced comprised 96 boars from a commercial synthetic sire line, born between 2005 and 2012. In order to ensure that the samples were representative of the line we selected unrelated animals from within the line using pedigree information and previously available information on genotypes. This sample exceeds the FAO Advisory Group on Animal Genetic Diversity guidelines which recommend sampling 25 unrelated individuals per breed [25].

**Figure 5:**
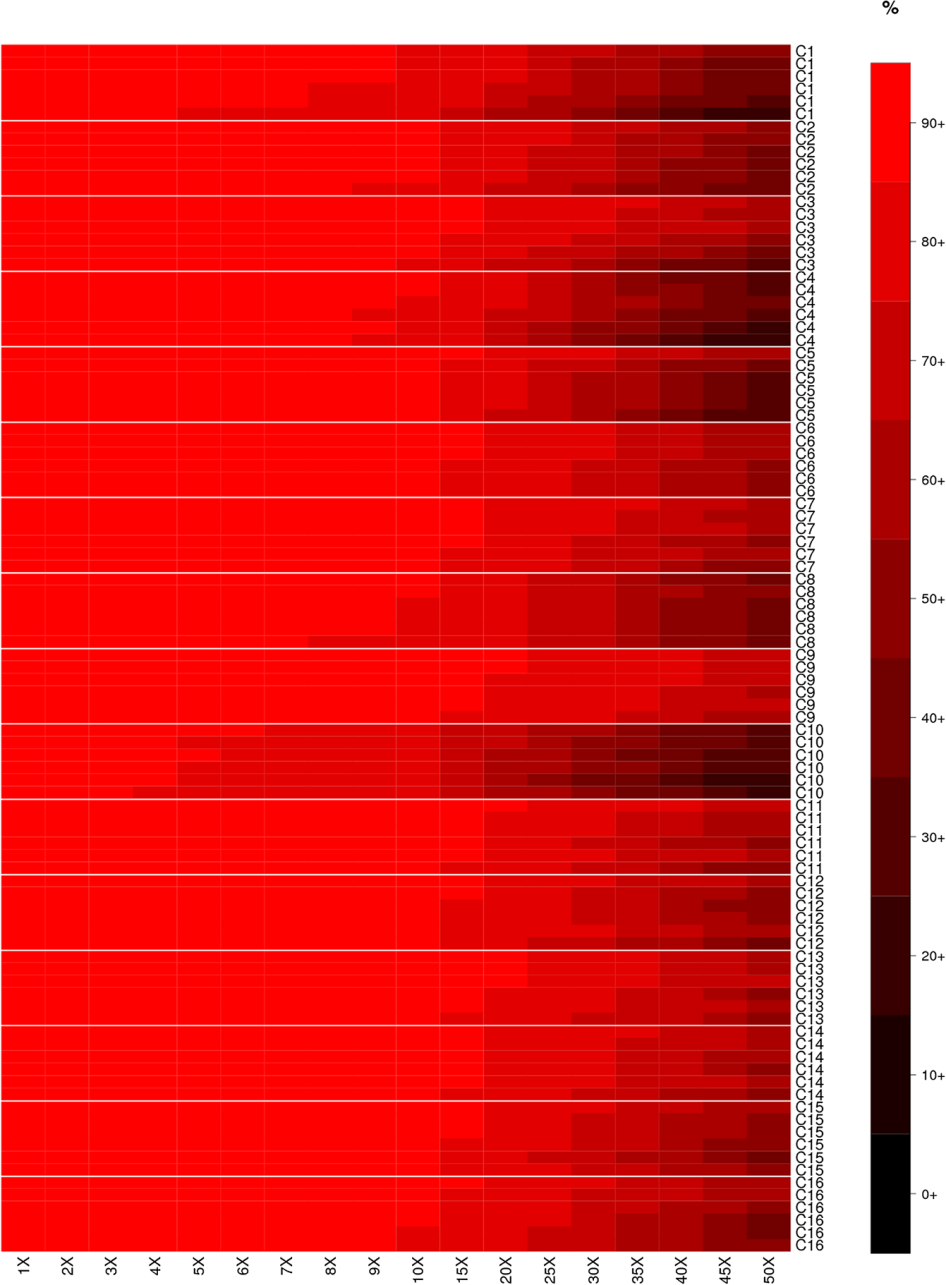
**Heatmap showing percentage of bases at a range of coverage levels for all 96 samples and 16 capture pools.**

Summary statistics for data volumes, mapping and coverage of the target are given in Table 1, and full results are given in Additional file 2: Table S2. Between 4.43 and 11.21 Gigabases of sequence data were produced for each of the 96 samples. Of the 96 samples, 72 achieved at least 90% of the target bases covered to at least a depth of 10X. The percentage of reads mapping to the genome was reasonably high (mean 89.62%), and of those that mapped, a mean of 67.75% overlapped with the target region. One goal for the future development of the design will be to improve both of those statistics. All samples achieved a PCR duplicate rate of less than 10%, with a mean of 3.78%. These values compare favourably with a previously published evaluation of human exome capture kits [26].Figure 5 shows a heatmap of the percentage of bases covered at a range of depths, from 1X to 50X. Clearly there is variation in efficiency of capture; C10 for example shows a decrease in coverage starting at 4X, whereas the same dip for capture C9 occurs at 15X.

**Table 1:** **Summary statistics for sequence volume, mapping and coverage**

Stat	Read pairs	Gbp	% mapped	% total on target	% mapped on target	% duplicates	1X	10X	30X	40X
Min	22141118	4.43	83.41	51.27	59.45	1.13	94.96	81.42	48.26	33.21
Max	56000083	11.20	93.96	68.64	73.28	9.48	98.07	94.29	86.99	82.49
Mean	38415939	7.68	89.62	60.74	67.75	3.78	97.13	91.11	75.02	64.33

Summary statistics from the exome sequencing of 96 pigs, showing minimum, maximum and mean values for a variety of sequence, mapping and coverage statistics. The columns in order represent: the statistic calculated; number of read pairs; gigabases sequenced; percentage of reads mapped; percentage of total reads overlapping with the target region; percentage of the mapped reads overlapping with the target; estimate of percent PCR duplicates; percent of bases in target covered at 1X; percent of bases in target covered at 10X; percent of bases in target covered at 30X; percent of bases in target covered at 40X.

### Variant discovery

Using published pipelines for variant discovery with GATK [22, 27] (see Methods) we predicted 237,334 high confidence single nucleotide polymorphisms (SNPs) present in at least one of the 96 pigs, present at 236,530 unique loci. 199,170 (83.9%) of the predictions have existing records in dbSNP. 236,608 (99.7%) of the SNPs are within our target region, and 173,849 (73.2%) overlap at least one exon in the current Ensembl genebuild. Of these SNPs, 93.8% were detected in more than one animal. 167,201 (70.4%) overlap with at least one exon of a protein-coding gene (including both UTR and CDS exons). Of the 167,201 SNPs within protein-coding genes, 71,460 (42.7%) overlap non-coding exons and 95,741 (57.3%) overlap a protein-coding region. Finally, of the 95,741 predicted SNPs within protein-coding exons, 60,732 (63.4%) are synonymous mutations, and 34,092 (35.6%) have an effect on the protein sequence. Of the 34,092 non-synonymous SNPs, 26,801 of these are known in dbSNP (78.6%) and 7291 are novel (21.4%). 399 SNPs are predicted to introduce a premature stop codon. Of these, 239 (60%) have an existing record in dbSNP and 160 (40%) are novel. The ratio of synonymous to non-synonymous mutations is very similar to what has been reported in humans and by analogy, around 10-20% of the non-synonymous variants are likely to adversely affect protein function [28, 29]. We have submitted all 237,334 SNPs to dbSNP.

Insertions and deletions (indels) were also predicted using the GATK pipeline. We predicted 28,976 indels, 28115 (97%) of which overlapped with our target region, and 18,471 (63.4%) of which overlapped with at least one exon in the current Ensembl genebuild. 17,503 (60.4%) overlapped an exon from a protein-coding gene. Of the 17,503 indels within protein coding genes, 13,352 (76.3%) overlap non-coding exons and 4,151 (23.7%) overlap a protein-coding region. Of these 4,151 protein-coding indels, 3386 are predicted to cause a frameshift in the protein sequence, 612 are in-frame indels and the remainder disrupt splice sites.

## Conclusions and discussion

In this paper we describe the first exome sequencing of pigs. We show that there remain large differences in the size of the predicted protein-coding exome between the published pig genome and those of human and mouse. The missing coding information can be attributed to two main factors: (i) regions of the pig genome that are not yet sequenced, or are poorly assembled; and (ii) gaps in the protein and RNA evidence available for genome annotation of the pig.

By adding EST evidence, we were able to increase the size of the target exome. Not all of the EST evidence will represent protein-coding genes, we have used the data simply to define “expressed regions”. The aligned ESTs do not necessarily make valid gene models (for example, the order of alignments (HSPs) in the EST may differ from the order in the genome). Such inconsistencies in the order of HSPs between EST/cDNA sequences and the genome sequences, which are probably the result of errors in short to medium range order and orientation of contigs in the genome assembly, could account for some the gaps in the Ensembl gene build. Despite these concerns, the ESTs provide at least some evidence that those portions of the genome are transcribed; many of the regions overlap or are close to existing annotated genes.

RNA-Seq data will become an increasingly important resource for genome annotation, in the pig and other genomes. A combination of exome-capture, RNA-Seq data and ab initio gene predictions (reviewed in [30]) will be used to further refine and capture exon boundaries and gene structures for individual protein-coding loci. In this paper we focus on the protein-coding exome. The annotation of the non-coding exome within the pig lags significantly behind human and mouse and few tools currently exist to predict the effects of variation in non-coding expressed DNA. As more data are published in both of these areas, we anticipate the inclusion of non-coding RNA genes in the exome capture.

Variants discovered by exome sequencing or whole genome re-sequencing experiments should be considered putative until confirmed by an independent assay or technology. The problems of variant discovery from current high throughput sequencing technologies are well documented [31, 32]. We have used published best practice for variant calling and filtering using GATK, however the number of variants we report in this paper is likely to be inflated. Variants from exome sequencing should be subject to further filters, depending on the nature of the experiment. In particular, insertions and deletions are problematic, and in a recent study to find loss-of-function variants in the human genome [33], the authors applied stringent filters and removed more than 50% of the candidate variants. Despite these problems, error rates tend to decline with higher coverage and can be less than 1%, depending on the software used [34].

The pig exome sequencing produced data of comparable quality to published studies in humans, with good coverage and on-target rates and low PCR-duplication. The HiSeq 2500 platform is capable of producing 42-45 Gb of sequence data per lane, therefore it is possible to multiplex 6 exome samples per lane. At that level (7.5 Gb of raw data per sample), exome sequencing will be approximately one third of the cost of whole genome sequencing to a depth of 10X. The choice of technology will vary depending on experimental question – the higher depth of exome sequencing provides greater power to detect variants, and cheaper costs allow researchers to process more samples whereas whole genome sequencing may uncover variants in non-exome regions, for example regulatory regions. With high throughput approaches to the identification of functional elements such as enhancers and promoters [35, 36], it may be possible to expand the exome-capture designs to include regulatory regions whilst retaining the cost advantages.

Exome capture experiments in the domestic pig will enable the rapid and cheap discovery of mutations relevant to a range of important traits, and facilitate the eradication of harmful mutations. In addition, as the pig is an excellent biomedical model for the study of human disease traits, much of the information on known disease-causing variants in the human exome can be used to inform model-based studies involving the pig. This combined with new methods for genome editing in livestock [37] will lead to a new and exciting era in livestock and human disease research. Combined with in-depth knowledge of gene expression [38], the variants identified by exome sequencing will prove to be a valuable resource for both agricultural and medical research.

## Methods

### Animals

All of the animals involved in this study were raised under conventional pig breeding and production conditions and were not subjected to any experimental procedures. DNA was prepared from piglet tail clips which is a conventional husbandry practice to prevent tail biting. These DNA samples were not collected specifically for this study, but rather were collected for identification and genetic diagnosis purposes.

### Defining the capture region

The Ensembl [39] gene annotations for the pig from release 71 were used as a starting point for the design, corresponding to assembly Sscrofa10.2 [12] and the May 2012 genebuild (patched Oct 2012). For comparison, Ensembl release 71 of both human and mouse was used, with assembly GRCh37.p10, genebuild April 2011 (patched Feb 2013) for human and assembly GRCm38.p1, genebuild Jul 2012 (patched Jan 2013) for mouse.

The file Sus_scrofa.Sscrofa10.2.71.gtf.gz was downloaded from the Ensembl ftp site, and the lengths of non-overlapping genomic regions corresponding to exons of protein-coding genes were summed. Having found that the pig “exome” is smaller than that of human and mouse, ESTs from build 42 of UniGene [21] Sus scrofa were mapped to the pig genome. The file Ssc.seq.uniq, representing the longest, best quality single sequence from each cluster, was downloaded from the NCBI FTP site, and used as input for NCBI BLASTN [40]. High-scoring segment pairs (HSPs) at least 50 bp in length and >90% identical were chosen; HSPs that mapped more than 200 times in the genome filtered out; and overlapping HSPs merged. The resulting regions summed to 22.5 Mb (mega-bases). These regions were merged with the Ensembl gene annotation using BEDTools [41]. The final set of target genomic regions sums to 58.1 Mb.

### Design of probes

The exome capture kits were purchased from Roche Nimblegen as the SeqCap EZ Developer Library product. The exome capture region, designated “ensembl + ests”, was provided to Roche Nimblegen and liquid capture probes designed according to their standard protocols. Using probes unique within the genome, that is probes that map exactly once in the genome, covers only 80% of the target regions; whereas using probes that map up to 5 times in the genome covers approximately 98.4% of the target regions. The latter set of probes was chosen and used in every downstream analysis. SSAHA was used to match probes to the genome, using the following parameters: minPrint = 35 (The minimum number of matching bases or residues that must be found in the query and subject sequences before they are considered as a match and thus printed); maxGap = 5 (Maximum gap allowed between successive hits for them to count as part of the same match); maxInsert = 5 (Maximum number of insertions/deletions allowed between successive hits for them to count as part of the same match); wordLength = 12 (Size in base pairs of the words used to form the hash table); and stepLength = 1 (Number of base pairs gap between words used to produce hash table).

### Capture, sequencing and bioinformatics

A total of 96 individual pig samples were sequenced. Initially 96 genomic DNA sequencing libraries were prepared using the Illumina Truseq DNA LT sample prep kit. Three micrograms of genomic DNA for each sample was sheared using a Covaris E220 instrument (LGC Genomics Ltd., Hoddesdon, UK) and purified with AMPure XP beads (Beckman Coulter Ltd, UK). Fragmentation quality was verified by running 1 μl of the sample on an Agilent D1K ScreenTape (Agilent Technologies Ltd. UK.). The remainder of each sample was used to construct the basic library. The end repair, A-tail and adapter ligation were carried out as specified in the Illumina Truseq DNA sample preparation protocol. The quality of each library was assessed using Agilent D1K High sensitivity ScreenTape to determine the size distribution for each library. Each Library was subject to precapture PCR following the Nimbelgen SeqCap EZ library preparation protocol. The cycling conditions were as follows: initial denaturation at 94°C for 10 minutes; 8 cycles of 94°C for 10 seconds, 60°C for 70 seconds and 72°C for 45 seconds; final extension at 72°C for 5 minutes, and cooling to 10°C until further use. The PCR products were purified with AMPure XP bead and analysed on a ScreenTape D1K (Agilent). The yield of each library was quantified using the Quant-iT™ PicoGreen® dsDNA kit (Life Technologies, Paisley, UK). Samples were pooled randomly in groups of six by mixing 200 ng of DNA for each library to form independent pools. One microgram of each pool was separately prepared for hybridization with the capture oligomers; the hybridizations were carried out at 47°C for 70 hours and the product captured using Streptavidin M-270 Dynabeads (Invitrogen, Carlsbad, CA, USA) according to the NimbleGen SeqCap EZ protocol. The captured DNA bound to the beads was subjected to LM-PCR as outlined in the Nimbelgen SeqCap EZ protocol except that the number of cycles was reduced to 10. The final PCR product was cleaned up using Ampure Beads and the quality of the captured DNA sequencing library assessed.

Each capture pool was analysed using the Agilent D1K ScreenTape for the size distribution of the captured DNA. Each pool was quantified by qPCR using the KAPA Library Quant Kits (KapaBiosystems; Anachem Ltd, beds, UK). All pools were diluted to a concentration of 10 nM for storage. The libraries were diluted to a concentration of 14 pM and loaded onto Illumina V3 paired end flow cell using the cBot (Illumina) according to the recommended protocol. Within each lane, each library was assigned unique Illumina adapter indices. Each pool was then sequenced in a single lane of the Illumina HiSeq 2500 system, generating 101 bp paired-end data.

Sus scrofa (assembly version 10.2) was downloaded from Ensembl (release 70) and used as the reference genome. The paired-end reads for each of the 96 samples were pre-processed in order to discard any pair in which at least one read contains one or more ambiguous bases. All resulting read pairs were then aligned against the pig reference genome using BWA [42]. SAMtools [43] was used to index/sort BAM files and index the reference genome. PCR and optical duplicates were removed from all alignments (BAM files) using the Picard MarkDuplicates tool (http://picard.sourceforge.net). The DepthofCoverage utility of the Genome Analysis Toolkit [22] was used to calculate the per-base depth of coverage in the exome capture targeted regions in each alignment file.

Variant (SNPs and indels) calling and genotyping across all 96 samples was performed with GATK following the general guidelines for whole exome sequencing [27]. Local re-alignment around indels was performed on alignments lacking duplicates using the following GATK tools: RealignerTargetCreator, IndelRealigner and FixMateInformation. Base quality score recalibration (GATK BaseRecalibrator) was then applied using the known Sus scrofa 10.2 variants (VCF) from Ensembl release 72 as the known sites. Variant calling was performed using GATK UnifiedGenotyper with downsampling to 10X coverage (option -dcov 100) threshold at which at least 90% of the target bases are covered for most samples (72/96). The results were filtered using GATK VariantFiltration with the following parameters: filter out variant calls if located within a cluster where three or more calls are made in a 10 bp window [clusterWindowSize 10]; filter out variant if there are at least four alignments with a mapping quality of zero (MQ0) and if the proportion of alignments mapping ambiguously corresponds to 1/10^th^ of all alignments [MQ0 > =4 && ((MQ0/(1.0 * DP)) > 0.1)], DP: total (unfiltered) depth over all samples; filter out variants which are covered by less than 5 reads [DP < 5]; filter out variants having a low quality score [Q < 50]; filter out variants with low variant confidence over unfiltered depth of non-reference samples (QD) [QD < 1.5]; filter out variants based on strand bias using Fisher's exact test: FS > 60.0 for SNP calling, FS > 200.0 for indel calling.

The Variant Effect Predictor tool from Ensembl [44] was used to identify the consequences of all variants in each sample.

### Availability of supporting data

The pig exome design is commercially available through Roche Nimblegen, design name 130104_Sscr_10_2_MW_EZ_HX1. The authors will be working on continuous improvements to this design; therefore scientists interested in pig exome research are encouraged to contact us. The SNPs discovered from exome sequencing of the 96 pigs in this study are available in dbSNP, ss numbers 1026566678–1026801732.

## Electronic supplementary material

Additional file 1: Table S1: Additional ESTs not found in the genome. A list of EST accessions and descriptions which do not align to the current pig reference genome, but which represent important genes we have added to the capture. (XLSX 16 KB)

Additional file 2: Table S2: Full statistics for the exome capture results. Full statistics describing the exome sequencing results, including number of reads, gigabases per sample, percentage mapped, percentage on target, percentage duplicates and percentage of bases covered at 1X, 10X, 30X and 40X. (XLSX 25 KB)
